# 1,3,5-Tris(4-bromo­phen­yl)-1,3,5-triazin­ane di­chloro­methane monosolvate

**DOI:** 10.1107/S1600536813013743

**Published:** 2013-05-25

**Authors:** Mahmoud Chebbah, Ahcene Bouchemma, Sofiane Bouacida, Leila Lefrada, Mustapha Bouhenguel

**Affiliations:** a Laboratoire de Chimie Appliquée et Technologie des Matériaux LCATM, Université Oum El Bouaghi, Algeria; b Département Sciences de la Matière, Faculté des Sciences Exactes et Sciences de la Nature et de la Vie, Université Oum El Bouaghi, Algeria; c Unité de Recherche de Cimie de l’Environnement et Moléculaire Structurale, CHEMS, Faculté des Sciences Exactes, Université Constantine 25000, Algeria

## Abstract

In the main mol­ecule of the title compound, C_21_H_18_Br_3_N_3_·CH_2_Cl_2_, the triazinane ring adopts a chair conformation with three 4-brom­ophenyl substituents, two in diaxial positions and the third in an equatorial arrangement (eaa). The torsion angles around the N—C bonds in the triazinane ring are in the range 55.6 (5)–60.1 (5)°. The structure can be described as being built up of alternating layers along the *b* axis with the CH_2_Cl_2_ solvent mol­ecules sandwiched between these layers. No classical hydrogen-bonding inter­actions are observed in the crystal structure.

## Related literature

For the conformations of 1,3,5-triaryl derivatives of 1,3,5-tri­aza­cyclo­hexane, see: Wellington & Tollens (1885 ▶); Bouchemma *et al.* (1988 ▶); Adam *et al.* (1993 ▶); Gilardi *et al.* (2003 ▶). 
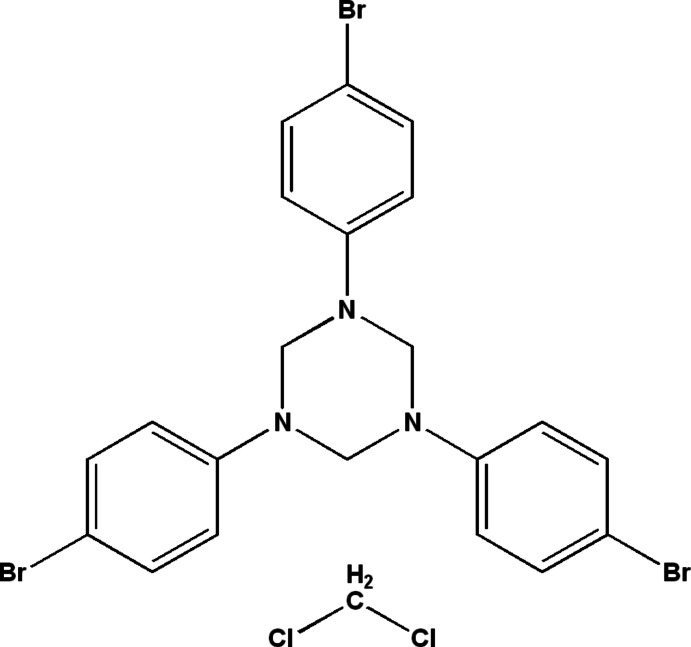


## Experimental

### 

#### Crystal data

C_21_H_18_Br_3_N_3_·CH_2_Cl_2_
*M* *_r_* = 637.04
Triclinic, 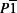

*a* = 6.0588 (2) Å
*b* = 14.3762 (6) Å
*c* = 15.1617 (6) Å
α = 65.323 (3)°
β = 89.759 (2)°
γ = 80.259 (2)°
*V* = 1179.46 (8) Å^3^
*Z* = 2
Mo *K*α radiation
μ = 5.37 mm^−1^
*T* = 295 K
0.24 × 0.24 × 0.08 mm

#### Data collection

Nonius KappaCCD diffractometer
Absorption correction: multi-scan (Blessing, 1995 ▶) *T*_min_ = 0.274, *T*_max_ = 0.467
13332 measured reflections
5637 independent reflections
3505 reflections with *I* > 2σ(*I*)
*R*_int_ = 0.078

#### Refinement

*R*[*F* ^2^ > 2σ(*F* ^2^)] = 0.060
*wR*(*F* ^2^) = 0.145
*S* = 1.09
5637 reflections
271 parameters
H-atom parameters constrained
Δρ_max_ = 0.67 e Å^−3^
Δρ_min_ = −0.96 e Å^−3^

### 

Data collection: *COLLECT* (Nonius, 2000) ▶; cell refinement: *SCALEPACK* (Otwinowski & Minor, 1997 ▶); data reduction: *DENZO* (Otwinowski & Minor, 1997 ▶) and *SCALEPACK*; program(s) used to solve structure: *SIR2002* (Burla *et al.*, 2005 ▶); program(s) used to refine structure: *SHELXL97* (Sheldrick, 2008 ▶); molecular graphics: *ORTEP-3 for Windows* (Farrugia, 2012 ▶) and *DIAMOND* (Brandenburg & Berndt, 2001 ▶); software used to prepare material for publication: *WinGX* (Farrugia, 2012 ▶).

## Supplementary Material

Crystal structure: contains datablock(s) global, I. DOI: 10.1107/S1600536813013743/bq2386sup1.cif

Structure factors: contains datablock(s) I. DOI: 10.1107/S1600536813013743/bq2386Isup2.hkl

Supplementary material file. DOI: 10.1107/S1600536813013743/bq2386Isup3.cml

Additional supplementary materials:  crystallographic information; 3D view; checkCIF report

## Figures and Tables

**Fig. 1.**
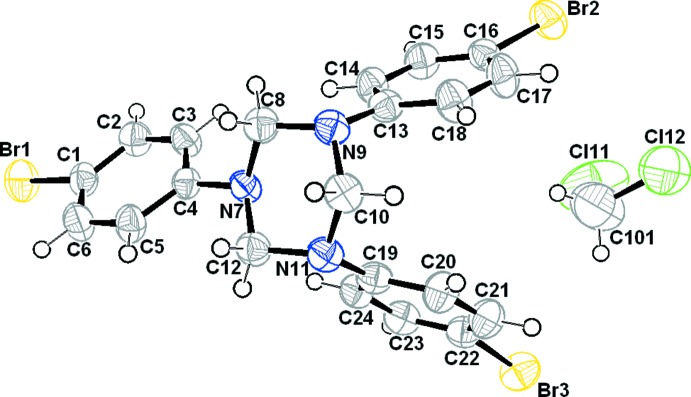
The structure of the title compound with the atomic labeling scheme. Displacements are drawn at the 50% probability level.

**Fig. 2.**
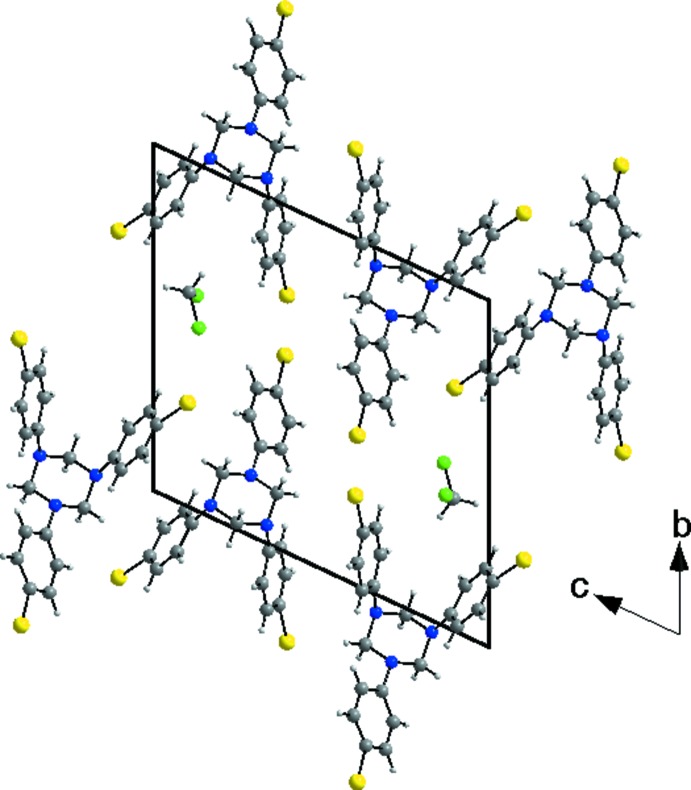
A diagram of the layered crystal packing in (I), viewed down the a axis.

## supplementary crystallographic information

### Comment

A variety of chair, twist-boat and boat conformations can be considered for 1,3,5-triazacyclohexanes with a pyramidal arrangement of bonds at the N atoms. Four types of chair conformation. eee, eea, eaa, and aaa; where e is equatorial and a is axial, are possible and each of these conformations results in axial interactions involving substituents or lone pair of electrons on the N atoms. X-ray investigation of 1,3,5-triazacylohexane of 1,3,5-trialkyl and 1,3,5-triarylderivatives of 1,3,5-triazacyclohexane have consistently found the expected chair conformation with pyramidal arrangement of bonds at N atoms (Wellington & Tollens, 1885; Bouchemma *et al.*, 1988; Adam *et al.*, 1993; Gilardi *et al.*, 2003). In the course of our studies in similar compounds we report here a conformation and crystal structure a new derivate of l,3,5-triazacylohexane, it is the product of a condensation reaction between 4-bromoaniline and formaldehyde. The molecular geometry and the atom-numbering scheme of (I) are shown in Fig. 1. The 1,3,5-tris(*p*-bromorophenyl)-l,3,5-triazacylohexane, adopts a chair conformation with two *p*-bromophenyl substituents situated in axial positions and a third in equatorial agreement (eaa). The structure can be described as alternating layers parallel to (010)planes, along the *b* axis and the dichloromethane solvent molecules are sandwiched between these layers (Fig.2). The packing of (I) is stabilized by a Van Der Waals interactions which form a three-dimensional network. No classical hydrogen bond was found.

### Experimental

To a solution of *p*-bromoaniline (25 mmol) in ethanol (10 ml), was added formaldehyde (5 ml, 37% aqueous solution). Stirring was then maintained at 25°C for 12 h. The precipitate thus formed was then collected and washed with diethyl ether. The residue was crystallized from dichloromethane.

### Refinement

All non-H atoms were refined with anisotropic atomic displacement parameters. All H atoms were localized on Fourier maps but introduced in calculated positions and treated as riding on their parent C atom, with C—H distances of 0.93 Å (C_aromatic_) and 0.97 Å (C_methylene_) and with *U*_iso_(H) = 1.2 *U*_eq_(C_aromatic_ and C_methylene_).

### Crystal data

**Table:**

C_21_H_18_Br_3_N_3_·CH_2_Cl_2_	*Z* = 2
*M**_r_* = 637.04	*F*(000) = 624
Triclinic, *P*1	*D*_x_ = 1.794 Mg m^−^^3^
Hall symbol: -P 1	Mo *K*α radiation, λ = 0.71073 Å
*a* = 6.0588 (2) Å	Cell parameters from 13332 reflections
*b* = 14.3762 (6) Å	θ = 1.5–28.5°
*c* = 15.1617 (6) Å	µ = 5.37 mm^−^^1^
α = 65.323 (3)°	*T* = 295 K
β = 89.759 (2)°	Prism, colourless
γ = 80.259 (2)°	0.24 × 0.24 × 0.08 mm
*V* = 1179.46 (8) Å^3^	

### Data collection

**Table:**

Nonius KappaCCD diffractometer	3505 reflections with *I* > 2σ(*I*)
Graphite monochromator	*R*_int_ = 0.078
ω + Phi scan	θ_max_ = 28.5°, θ_min_ = 3.4°
Absorption correction: multi-scan (Blessing, 1995)	*h* = −8→7
*T*_min_ = 0.274, *T*_max_ = 0.467	*k* = −18→19
13332 measured reflections	*l* = −17→19
5637 independent reflections	

### Refinement

**Table:**

Refinement on *F*^2^	Primary atom site location: structure-invariant direct methods
Least-squares matrix: full	Secondary atom site location: difference Fourier map
*R*[*F*^2^ > 2σ(*F*^2^)] = 0.060	Hydrogen site location: inferred from neighbouring sites
*wR*(*F*^2^) = 0.145	H-atom parameters constrained
*S* = 1.09	*w* = 1/[σ^2^(*F*_o_^2^) + (0.0428*P*)^2^ + 1.5896*P*] where *P* = (*F*_o_^2^ + 2*F*_c_^2^)/3
5637 reflections	(Δ/σ)_max_ = 0.001
271 parameters	Δρ_max_ = 0.67 e Å^−^^3^
0 restraints	Δρ_min_ = −0.96 e Å^−^^3^

### Special details

**Table:**

Geometry. All e.s.d.'s (except the e.s.d. in the dihedral angle between two l.s. planes) are estimated using the full covariance matrix. The cell e.s.d.'s are taken into account individually in the estimation of e.s.d.'s in distances, angles and torsion angles; correlations between e.s.d.'s in cell parameters are only used when they are defined by crystal symmetry. An approximate (isotropic) treatment of cell e.s.d.'s is used for estimating e.s.d.'s involving l.s. planes.
Refinement. Refinement of *F*^2^ against ALL reflections. The weighted *R*-factor *wR* and goodness of fit *S* are based on *F*^2^, conventional *R*-factors *R* are based on *F*, with *F* set to zero for negative *F*^2^. The threshold expression of *F*^2^ > σ(*F*^2^) is used only for calculating *R*-factors(gt) *etc*. and is not relevant to the choice of reflections for refinement. *R*-factors based on *F*^2^ are statistically about twice as large as those based on *F*, and *R*- factors based on ALL data will be even larger.

### Fractional atomic coordinates and isotropic or equivalent isotropic displacement parameters (Å^2^)

**Table:**

	*x*	*y*	*z*	*U*_iso_*/*U*_eq_	
Br1	0.18017 (11)	1.56642 (5)	0.60749 (5)	0.0660 (2)	
Br2	0.65521 (10)	0.70178 (5)	1.10267 (4)	0.0635 (2)	
Br3	0.63610 (10)	0.74321 (5)	0.60175 (5)	0.05954 (19)	
C1	0.4134 (9)	1.4501 (4)	0.6332 (4)	0.0435 (12)	
C2	0.6030 (10)	1.4347 (4)	0.6909 (4)	0.0534 (14)	
H2	0.623	1.4834	0.7142	0.064*	
C3	0.7646 (9)	1.3449 (4)	0.7140 (4)	0.0472 (12)	
H3	0.894	1.3345	0.7522	0.057*	
C4	0.7355 (8)	1.2711 (4)	0.6808 (3)	0.0365 (10)	
C5	0.5461 (8)	1.2912 (4)	0.6201 (4)	0.0442 (12)	
H5	0.5272	1.2437	0.5952	0.053*	
C6	0.3851 (9)	1.3797 (4)	0.5958 (4)	0.0482 (12)	
H6	0.2593	1.392	0.5549	0.058*	
N7	0.8851 (7)	1.1745 (3)	0.7092 (3)	0.0390 (9)	
C8	1.0412 (9)	1.1433 (4)	0.7946 (4)	0.0447 (12)	
H8A	1.1555	1.1865	0.7776	0.054*	
H8B	0.9603	1.1536	0.8461	0.054*	
N9	1.1476 (7)	1.0340 (3)	0.8290 (3)	0.0427 (10)	
C10	1.2702 (8)	1.0189 (4)	0.7518 (4)	0.0441 (12)	
H10A	1.3363	0.9458	0.7741	0.053*	
H10B	1.3913	1.0582	0.7372	0.053*	
N11	1.1234 (7)	1.0525 (3)	0.6625 (3)	0.0425 (10)	
C12	1.0132 (9)	1.1595 (4)	0.6313 (4)	0.0442 (12)	
H12A	0.9119	1.1797	0.5744	0.053*	
H12B	1.1247	1.2041	0.6131	0.053*	
C13	1.0220 (8)	0.9572 (4)	0.8855 (3)	0.0395 (11)	
C14	0.8000 (8)	0.9826 (4)	0.9047 (4)	0.0439 (12)	
H14	0.7244	1.0513	0.8752	0.053*	
C15	0.6918 (8)	0.9057 (4)	0.9678 (4)	0.0471 (12)	
H15	0.5443	0.9231	0.9808	0.057*	
C16	0.7999 (8)	0.8052 (4)	1.0105 (4)	0.0445 (12)	
C17	1.0182 (9)	0.7775 (4)	0.9902 (4)	0.0514 (13)	
H17	1.0903	0.7082	1.0176	0.062*	
C18	1.1266 (8)	0.8542 (4)	0.9287 (4)	0.0481 (13)	
H18	1.2741	0.8361	0.916	0.058*	
C19	1.0037 (8)	0.9794 (4)	0.6553 (3)	0.0402 (11)	
C20	1.1126 (9)	0.8785 (4)	0.6758 (4)	0.0529 (14)	
H20	1.2624	0.8579	0.6996	0.064*	
C21	1.0056 (9)	0.8089 (4)	0.6619 (4)	0.0548 (14)	
H21	1.0819	0.7418	0.6767	0.066*	
C22	0.7831 (8)	0.8385 (4)	0.6256 (4)	0.0440 (12)	
C23	0.6693 (9)	0.9354 (4)	0.6074 (4)	0.0491 (13)	
H23	0.5183	0.9541	0.5854	0.059*	
C24	0.7774 (8)	1.0066 (4)	0.6214 (4)	0.0447 (12)	
H24	0.6984	1.0729	0.6081	0.054*	
Cl11	0.4359 (4)	0.6198 (3)	0.8705 (2)	0.1647 (16)	
Cl12	0.8927 (4)	0.5297 (2)	0.86456 (17)	0.1056 (7)	
C101	0.7167 (16)	0.6197 (8)	0.8929 (7)	0.118 (3)	
H10C	0.749	0.6886	0.8545	0.142*	
H10D	0.7454	0.6037	0.9611	0.142*	

### Atomic displacement parameters (Å^2^)

**Table:**

	*U*^11^	*U*^22^	*U*^33^	*U*^12^	*U*^13^	*U*^23^
Br1	0.0620 (4)	0.0496 (4)	0.0824 (5)	0.0053 (3)	0.0044 (3)	−0.0299 (3)
Br2	0.0582 (4)	0.0680 (4)	0.0602 (4)	−0.0218 (3)	0.0080 (3)	−0.0194 (3)
Br3	0.0568 (4)	0.0626 (4)	0.0712 (4)	−0.0116 (3)	−0.0024 (3)	−0.0396 (3)
C1	0.045 (3)	0.035 (3)	0.049 (3)	−0.006 (2)	0.011 (2)	−0.018 (2)
C2	0.060 (4)	0.042 (3)	0.064 (4)	−0.011 (3)	0.001 (3)	−0.028 (3)
C3	0.045 (3)	0.039 (3)	0.058 (3)	−0.011 (2)	−0.002 (2)	−0.020 (2)
C4	0.038 (2)	0.032 (2)	0.039 (3)	−0.010 (2)	0.0048 (19)	−0.014 (2)
C5	0.045 (3)	0.044 (3)	0.050 (3)	−0.008 (2)	−0.002 (2)	−0.026 (2)
C6	0.043 (3)	0.048 (3)	0.054 (3)	−0.009 (2)	−0.004 (2)	−0.022 (3)
N7	0.041 (2)	0.039 (2)	0.042 (2)	−0.0051 (18)	0.0024 (17)	−0.0227 (18)
C8	0.045 (3)	0.044 (3)	0.046 (3)	−0.007 (2)	−0.005 (2)	−0.021 (2)
N9	0.035 (2)	0.048 (3)	0.045 (2)	−0.0025 (19)	−0.0031 (17)	−0.0218 (19)
C10	0.030 (2)	0.051 (3)	0.054 (3)	−0.009 (2)	0.005 (2)	−0.024 (2)
N11	0.037 (2)	0.047 (3)	0.045 (2)	−0.0044 (19)	0.0068 (17)	−0.023 (2)
C12	0.042 (3)	0.044 (3)	0.047 (3)	−0.011 (2)	0.011 (2)	−0.018 (2)
C13	0.032 (2)	0.048 (3)	0.039 (3)	−0.005 (2)	−0.0040 (19)	−0.019 (2)
C14	0.035 (3)	0.049 (3)	0.049 (3)	−0.001 (2)	0.002 (2)	−0.024 (2)
C15	0.035 (3)	0.057 (3)	0.052 (3)	−0.004 (2)	0.001 (2)	−0.027 (3)
C16	0.041 (3)	0.053 (3)	0.043 (3)	−0.012 (2)	0.001 (2)	−0.022 (2)
C17	0.044 (3)	0.048 (3)	0.055 (3)	−0.003 (2)	0.000 (2)	−0.017 (3)
C18	0.030 (3)	0.048 (3)	0.059 (3)	0.003 (2)	−0.001 (2)	−0.019 (3)
C19	0.037 (3)	0.044 (3)	0.039 (3)	−0.004 (2)	0.007 (2)	−0.018 (2)
C20	0.038 (3)	0.052 (3)	0.070 (4)	0.001 (2)	−0.004 (2)	−0.030 (3)
C21	0.044 (3)	0.044 (3)	0.075 (4)	0.007 (2)	−0.005 (3)	−0.029 (3)
C22	0.045 (3)	0.048 (3)	0.043 (3)	−0.008 (2)	0.000 (2)	−0.022 (2)
C23	0.040 (3)	0.051 (3)	0.053 (3)	−0.003 (2)	−0.006 (2)	−0.020 (3)
C24	0.036 (3)	0.042 (3)	0.053 (3)	0.004 (2)	−0.002 (2)	−0.020 (2)
Cl11	0.0865 (17)	0.237 (4)	0.112 (2)	0.038 (2)	−0.0031 (14)	−0.043 (2)
Cl12	0.0865 (14)	0.1185 (18)	0.1040 (16)	0.0149 (13)	−0.0080 (11)	−0.0519 (13)
C101	0.115 (8)	0.137 (8)	0.117 (7)	−0.001 (6)	−0.005 (6)	−0.076 (7)

### Geometric parameters (Å, º)

**Table:**

Br1—C1	1.899 (5)	C12—H12A	0.97
Br2—C16	1.904 (5)	C12—H12B	0.97
Br3—C22	1.905 (5)	C13—C18	1.378 (7)
C1—C2	1.376 (8)	C13—C14	1.395 (7)
C1—C6	1.383 (7)	C14—C15	1.388 (7)
C2—C3	1.394 (7)	C14—H14	0.93
C2—H2	0.93	C15—C16	1.355 (7)
C3—C4	1.386 (7)	C15—H15	0.93
C3—H3	0.93	C16—C17	1.387 (7)
C4—C5	1.386 (7)	C17—C18	1.378 (7)
C4—N7	1.419 (6)	C17—H17	0.93
C5—C6	1.377 (7)	C18—H18	0.93
C5—H5	0.93	C19—C20	1.392 (7)
C6—H6	0.93	C19—C24	1.398 (7)
N7—C8	1.468 (6)	C20—C21	1.364 (8)
N7—C12	1.480 (6)	C20—H20	0.93
C8—N9	1.460 (6)	C21—C22	1.385 (7)
C8—H8A	0.97	C21—H21	0.93
C8—H8B	0.97	C22—C23	1.359 (7)
N9—C13	1.422 (6)	C23—C24	1.390 (7)
N9—C10	1.456 (6)	C23—H23	0.93
C10—N11	1.475 (6)	C24—H24	0.93
C10—H10A	0.97	Cl11—C101	1.736 (10)
C10—H10B	0.97	Cl12—C101	1.729 (9)
N11—C19	1.413 (7)	C101—H10C	0.97
N11—C12	1.444 (6)	C101—H10D	0.97
			
C2—C1—C6	120.9 (5)	H12A—C12—H12B	108
C2—C1—Br1	120.3 (4)	C18—C13—C14	118.3 (5)
C6—C1—Br1	118.8 (4)	C18—C13—N9	119.2 (4)
C1—C2—C3	119.1 (5)	C14—C13—N9	122.4 (4)
C1—C2—H2	120.5	C15—C14—C13	120.1 (5)
C3—C2—H2	120.5	C15—C14—H14	119.9
C4—C3—C2	121.1 (5)	C13—C14—H14	119.9
C4—C3—H3	119.5	C16—C15—C14	120.4 (5)
C2—C3—H3	119.5	C16—C15—H15	119.8
C3—C4—C5	118.1 (5)	C14—C15—H15	119.8
C3—C4—N7	123.2 (4)	C15—C16—C17	120.5 (5)
C5—C4—N7	118.7 (4)	C15—C16—Br2	119.9 (4)
C6—C5—C4	121.8 (5)	C17—C16—Br2	119.6 (4)
C6—C5—H5	119.1	C18—C17—C16	119.0 (5)
C4—C5—H5	119.1	C18—C17—H17	120.5
C5—C6—C1	119.0 (5)	C16—C17—H17	120.5
C5—C6—H6	120.5	C13—C18—C17	121.6 (5)
C1—C6—H6	120.5	C13—C18—H18	119.2
C4—N7—C8	116.2 (4)	C17—C18—H18	119.2
C4—N7—C12	116.0 (4)	C20—C19—C24	117.3 (5)
C8—N7—C12	108.5 (4)	C20—C19—N11	120.6 (4)
N9—C8—N7	110.4 (4)	C24—C19—N11	122.0 (5)
N9—C8—H8A	109.6	C21—C20—C19	121.8 (5)
N7—C8—H8A	109.6	C21—C20—H20	119.1
N9—C8—H8B	109.6	C19—C20—H20	119.1
N7—C8—H8B	109.6	C20—C21—C22	119.9 (5)
H8A—C8—H8B	108.1	C20—C21—H21	120.1
C13—N9—C10	117.9 (4)	C22—C21—H21	120.1
C13—N9—C8	117.9 (4)	C23—C22—C21	120.0 (5)
C10—N9—C8	109.5 (4)	C23—C22—Br3	120.0 (4)
N9—C10—N11	111.8 (4)	C21—C22—Br3	120.0 (4)
N9—C10—H10A	109.2	C22—C23—C24	120.4 (5)
N11—C10—H10A	109.2	C22—C23—H23	119.8
N9—C10—H10B	109.3	C24—C23—H23	119.8
N11—C10—H10B	109.3	C23—C24—C19	120.6 (5)
H10A—C10—H10B	107.9	C23—C24—H24	119.7
C19—N11—C12	119.7 (4)	C19—C24—H24	119.7
C19—N11—C10	117.5 (4)	Cl12—C101—Cl11	111.7 (6)
C12—N11—C10	110.1 (4)	Cl12—C101—H10C	109.3
N11—C12—N7	111.3 (4)	Cl11—C101—H10C	109.3
N11—C12—H12A	109.4	Cl12—C101—H10D	109.3
N7—C12—H12A	109.4	Cl11—C101—H10D	109.3
N11—C12—H12B	109.4	H10C—C101—H10D	107.9
N7—C12—H12B	109.4		
			
C6—C1—C2—C3	−1.9 (8)	C18—C13—C14—C15	1.6 (7)
Br1—C1—C2—C3	175.7 (4)	N9—C13—C14—C15	−173.9 (5)
C1—C2—C3—C4	−0.8 (8)	C13—C14—C15—C16	−0.6 (8)
C2—C3—C4—C5	3.0 (8)	C14—C15—C16—C17	−1.4 (8)
C2—C3—C4—N7	−174.3 (5)	C14—C15—C16—Br2	176.3 (4)
C3—C4—C5—C6	−2.5 (8)	C15—C16—C17—C18	2.4 (8)
N7—C4—C5—C6	174.9 (5)	Br2—C16—C17—C18	−175.4 (4)
C4—C5—C6—C1	−0.1 (8)	C14—C13—C18—C17	−0.6 (8)
C2—C1—C6—C5	2.4 (8)	N9—C13—C18—C17	175.0 (5)
Br1—C1—C6—C5	−175.2 (4)	C16—C17—C18—C13	−1.3 (9)
C3—C4—N7—C8	15.3 (7)	C12—N11—C19—C20	173.6 (4)
C5—C4—N7—C8	−162.0 (4)	C10—N11—C19—C20	−48.4 (6)
C3—C4—N7—C12	−114.0 (5)	C12—N11—C19—C24	−2.1 (7)
C5—C4—N7—C12	68.7 (6)	C10—N11—C19—C24	135.9 (5)
C4—N7—C8—N9	167.0 (4)	C24—C19—C20—C21	1.2 (8)
C12—N7—C8—N9	−60.1 (5)	N11—C19—C20—C21	−174.6 (5)
N7—C8—N9—C13	−78.9 (5)	C19—C20—C21—C22	0.6 (9)
C13—N9—C10—N11	81.6 (5)	C20—C21—C22—C23	−2.5 (9)
C8—N9—C10—N11	−56.9 (5)	C20—C21—C22—Br3	177.8 (4)
N9—C10—N11—C19	−86.2 (5)	C21—C22—C23—C24	2.5 (8)
C19—N11—C12—N7	84.4 (5)	Br3—C22—C23—C24	−177.8 (4)
C10—N11—C12—N7	−56.4 (5)	C22—C23—C24—C19	−0.6 (8)
C4—N7—C12—N11	−168.1 (4)	C20—C19—C24—C23	−1.2 (7)
C10—N9—C13—C18	52.7 (6)	N11—C19—C24—C23	174.6 (4)
C8—N9—C13—C18	−172.3 (4)	N7—C8—N9—C10	59.6 (5)
C10—N9—C13—C14	−131.9 (5)	C8—N7—C12—N11	58.9 (5)
C8—N9—C13—C14	3.1 (7)	N9—C10—N11—C12	55.6 (5)
